# The higher-order structure of early maladaptive schemas: A meta-analytical approach

**DOI:** 10.3389/fpsyt.2022.1053927

**Published:** 2022-12-01

**Authors:** Jens C. Thimm

**Affiliations:** ^1^Centre for Crisis Psychology, University of Bergen, Bergen, Norway; ^2^Department of Psychology, UiT The Arctic University of Norway, Tromsø, Norway

**Keywords:** early maladaptive schemas (EMS), schema domains, meta-analysis, confirmatory factor analysis, principal component analysis

## Abstract

**Background:**

Early maladaptive schemas (EMSs) are themes regarding oneself and one's relationship with others. In schema therapy, 18 EMSs are described that were initially proposed to be clustered in five domains. The current EMS model comprises four domains. However, empirical investigations into the grouping of EMSs have yielded divergent results. The purpose of the present study was to use a meta-analytical approach to examine the higher-order organization of EMSs.

**Methods:**

To be included in the statistical analyses, studies had to report the associations between all 18 EMSs using a form of the Young Schema Questionnaire. In a systematic literature review in PsycInfo, Embase, MEDLINE, Web of Science, and Google scholar, 27 studies were identified that reported the associations between EMSs in 33 independent samples (*N* = 13,958, *M*_age_ = 16–72.3 years, 64.0% female). The correlations between EMSs were pooled across samples and analyzed using confirmatory factor analysis (CFA) and principal component analysis (PCA).

**Results:**

The CFA results showed weak support for any of the previously suggested EMS domains. After PCA, four EMS domains were retained that closely resembled the theoretically proposed organization of EMSs. The four components showed fair to good congruence in the clinical and non-clinical subsamples. However, a model with three EMS domains showed a simpler structure.

**Discussion:**

The results suggest a need for further theoretical and empirical clarification of the higher-order structure of EMSs.

**Systematic review registration:**

https://osf.io/57wyz.

## Introduction

The concept of early maladaptive schemas (EMSs) is at the core of schema therapy (ST) (1), which is an integrative psychological treatment for personality pathology and recurrent or chronic emotional disorders. Treatment with ST has been shown to be effective for personality disorders [e.g., (2, 3)]. The effectiveness of ST has further been examined for a range of other psychiatric diagnoses, including, but not limited to, depression [e.g., (4)], anxiety disorders (5), and eating disorders [e.g., (6)]. Young (7) defines EMSs as “extremely stable and enduring themes that develop during childhood, are elaborated throughout an individual's lifetime and are dysfunctional to a significant degree” (p. 9). It is theorized in ST that EMSs originate from the frustration of core emotional needs such as love, acceptance, and autonomy due to abuse, neglect, or over-indulgence (1). Young (8) developed a list of EMSs that has evolved over time and currently comprises 18 EMSs (7). For example, defectiveness/shame refers to the feeling that one is defective and fundamentally flawed while entitlement/grandiosity involves the belief that one is superior to others and is entitled to privileges [for definitions of all 18 EMSs, see (1, 9)]. The Young Schema Questionnaire (YSQ) (8) is a self-report inventory for the assessment of EMSs that has been revised following revisions of the list of EMSs and that is at this time available as a 232-item questionnaire (10) and a 90-item short form (11).

Since the first version of the EMS list (8), EMSs have been grouped into clusters or domains. Initially, the five domains of impaired autonomy, disconnection, undesirability, restricted self-expression, and impaired limits were proposed (8). Subsequently, the five domains were reorganized and some domains were renamed, resulting in a model with the domains of disconnection and rejection, impaired autonomy and performance, impaired limits, other-directedness, and over-vigilance and inhibition (7). These domains reflect the frustration of the needs for secure attachment, autonomy, realistic limits, freedom to express needs, and spontaneity, respectively (7). The EMSs that form these five domains are shown in Table 1. Based on factor-analytic studies of the YSQ (22, 23), Young (12) and Bach et al. (19) revised the proposed organization of EMSs into a model with four higher-order domains, namely disconnection and rejection, impaired autonomy and performance, impaired limits, and excessive responsibility and standards (see Table 1). Notably, the most recently added EMSs (approval/recognition seeking, negativity/pessimism, and punitiveness) were not classified by Young (12) because their clustering with other EMSs was unclear.

**Table 1:** Proposed EMS organizations.

**EMS**	**Young (7)^a^**	**Young (12)^a^**	**Unoka et al. (13)^b^**	**Soygüt et al. (14)^a^**	**Saritas and Gencöz (15)^b^**	**Csukly et al. (16)^b^**	**Saariaho et al. (17)^b^**	**Saariaho et al. (17)^b^**	**Calvete et al. (18)^a^**	**Bach et al. (19)^a^**	**Yalcin et al. (20)^a^**	**Macik and Macik (21)^b^**
**Sample**	n/a	n/a	Clinical (*n* = 114)	Nonclinical (*n* = 1071)	Nonclinical (*n* = 356)	Clinical (*n* = 107)	Clinical (*n* = 268)	Nonclinical (*n* = 324)	Nonclinical (*n* = 952)	Mixed (*n* = 1049)	Mixed (*n* = 838)	Nonclinical (*n* = 2348)
**Assessment of EMSs**	n/a	n/a	extended YSQ-L2	YSQ-S3	YSQ-S3	extended YSQ-L2	extended YSQ-SF	extended YSQ-SF	YSQ-S3	YSQ-S3	YSQ-L3	YSQ-S3
**Method**	n/a	n/a	PCA	not reported	PCA	PCA	PAF	PAF	PCA	PCA	PAF	FA
**Factor extraction**	n/a	n/a	eigenvalue >1, loadings >.40	not reported	scree plot, eigenvalue, residual correlation matrix	eigenvalue >1, factor loading >0.4	scree test, eigenvalue >1, expl. variance 5-10%, ≥2 sign. loadings, interpretability	scree test, eigenvalue >1, expl. variance 5-10%, ≥2 sign. loadings, interpretability	scree test	parallel analysis, eigenvalue > 1, scree plot, ≥ 3 primary loadings	scree test, eigenvalues > 1	models with 1, 4, and 5 factors calculated
**Number of factors**	n/a	n/a	4	5	3	4	2	3	3	4	4	4
**Factor rotation**	n/a	n/a	Varimax	not reported	Varimax	Varimax	Promax	Promax	Varimax	Equamax	Promax	Varimax
**Abandonment/ instability**	DR	IA	Factor 1 (Factor 2)	IA	IA/OD	Factor 2 Factor 1	LO	END	IA	IA	D	Factor 1
**Mistrust/abuse**	DR	DR	Factor 1	DR	IL/ES (DR)	Factor 2 (Factor 3)	LO	LON	DR	DR	D (ED)	Factor 1 (Factor 2)
**Emotional deprivation**	DR	DR	Factor 1	DR	DR	Factor 3	LO	LON	DR	DR	D	Factor 2 (Factor 1)
**Defectiveness/ shame**	DR	DR	Factor 2 (Factor 1)	DR	DR (IA/OD)	Factor 1 (Factor 3)	LO	END	DR	DR	D	Factor 2 (Factor 1)
**Social isolation/ alienation**	DR	DR	Factor 1 (Factor 2)	DR	DR	Factor 3 (Factor 1)	LO	LON	DR	DR	D	Factor 2
**Dependence/ incompetence**	IA	IA	Factor 2	IA	IA/OD (DR)	Factor 1	LO	END	IA	IA	IA/US	Factor 1 (Factor 2)
**Vulnerability to harm or illness**	IA	IA	Factor 1 (Factor 3)	IA	IA/OD (IL/ES)	Factor 4	LO	END	IA	IA (DR)	ED	Factor 1
**Enmeshment/ undeveloped self**	IA	IA	Factor 2	IA	IA/OD	Factor 1	LO	END	IA	IA (ER)	IA/US	Factor 1
**Failure**	IA	IA	Factor 2	IA	DR (IA/OD)	Factor 1	LO	END	IA	IA	IA/US	Factor 1 (Factor 2)
**Entitlement/ grandiosity**	IL	IL	Factor 4	IL	IL/ES	Factor 2	EN	ENC	Factor III	IL	ED	Factor 3
**Insufficient self-control/ self-discipline**	IL	IL	Factor 1, Factor 4 (Factor 2)	IL	IL/ES	Factor 2 (Factor 1)	LO	END	IA	IL (IA)	ED (IA/US)	Factor 3 (Factor 1)
**Subjugation/ invalidation**	OD	IA	Factor 2		IA/OD	Factor 1 (Factor 4)	LO	END	IA	IA (ER)	IA/US	Factor 1 (Factor 2)
**Self-sacrifice**	OD	ER	Factor 3	OD	IA/OD (IL/ES)	Factor 4	EN	ENC	Factor III	ER	ER	Factor 4
**Approval-seeking/ recognition-seeking**	OD	Un-classified	Factor 2 (Factor 4)	UR	IL/ES	Factor 1 (Factor 4, Factor 2)	EN	ENC	Factor III	IL	IA/US (ER)	Factor 3
**Negativity/ pessimism**	OV	Un-classified	Factor 1 (Factor 3)	IA	IL/ES (IA/OD)	Factor 1 (Factor 3)	EN	END	Factor III	DR (IA, ER)	ED	Factor 1
**Emotional inhibition**	OV	DR	Factor 1	DR	DR	Factor 3 (Factor 1)	LO	LON	DR	DR	ED	Factor 2
**Unrelenting standards/ hypercriticalness**	OV	ER	Factor 3	UR	IL/ES	Factor 4	EN	ENC	Factor III	ER	ER	Factor 4 (Factor 3)
**Punitiveness**	OV	Un-classified	Factor 3 (Factor 4)	OD	IL/ES	Factor 2 (Factor 4)	EN	ENC	Factor III	ER (DR)	ED	Factor 1

a EMS organization by the author(s),

b EMS organization based on the highest factor loadings (secondary loadings ≥0.40 in brackets). D, Disconnection; DR, Disconnection and rejection; ED, Emotional dysregulation; ENC, Encumbered; END, Endangered; ER, Excessive responsibility; ES, Exaggerated standards; IA, Impaired autonomy; IL, Impaired limits; LO, Loser; LON, Lonely; OD, Other-directedness; OV, Overvigilance and inhibition; UR, Unrelenting standards; US, Undeveloped self.

Several studies have examined the higher-order structure of EMSs with exploratory factor analysis (EFA). Table 1 provides an overview of study results on the currently defined18 EMSs [for EFA studies on earlier EMS lists, see (18, 24)]. As shown in Table 1, different versions of the YSQ, factor-analytic methods, factor extraction criteria, and rotation methods were used in the studies, and the authors presented two (17) to five (14) EMS domains, with most researchers favoring three-factor [e.g., (18)] or four-factor solutions [e.g., (13, 19, 20)]. However, even if the same number of domains was proposed, the composition and interpretation of the domains varied between studies. For example, Bach et al. (19) found support for the four domains proposed by Young (12) using the YSQ-S3 and identified the approval/recognition seeking, negativity/pessimism, and punitiveness EMSs in the impaired limits, disconnection/rejection, and excessive responsibility and standards domains, respectively. In contrast, Yalcin et al. (20) suggested an emotional dysregulation domain in addition to the disconnection, impaired autonomy/underdeveloped self, and excessive responsibility/overcontrol domains based on findings with the YSQ-L3. In the Yalcin et al. (20) model, the emotional dysregulation domain is defined by the EMSs of entitlement, punitiveness, emotional inhibition, negativity/pessimism, and vulnerability to harm. The approval/recognition EMS is proposed to be part of the impaired autonomy/underdeveloped self domain (20). Thus the findings from EFA studies are inconclusive.

Other researchers have used confirmatory factor analysis (CFA) to investigate the organization of EMSs into domains [e.g., (18, 21, 24–31)]. Overall, weak support has been found for Young's (7) five-domain model. Except for the study by Young (7) and Calvete et al. (18) five domains fit the data poorly [e.g., (24, 27, 29)]. However, most studies also reported a poor fit for models with one to four factors [e.g., (21, 24, 30)]. Kriston et al. (24) found that a bifactorial model in which all YSQ items loaded on a single generic factor and first-order EMS factors showed the best fit in their data, but Saggino et al. (29) reported inadequate fit of this model in their study. Taken together, most CFA studies have failed to support any of the proposed domain models.

Consensus on the number and organization of higher-order EMS domains is important for theory development and research. Because the EMS domains are thought to be connected to different basic emotional needs, the number and content of EMS domains provide information about the number and specific emotional needs on which EMS theory is built in ST. Further, many studies focus on EMS domains instead of specific EMSs [e.g., (32–34)]. Agreement on the definitions of EMS domains will make study results easier to compare.

A limitation of individual studies on the organization of EMSs is that the results are influenced by the characteristics of the specific samples. The aim of the present study was to investigate the higher-order structure of EMSs by meta-analytically combining data on the associations between EMSs in different samples. Based on Young's (12) revised organization of EMSs, it was hypothesized that four domains will represent the data most adequately. However, no hypotheses were made regarding the content and definitions of the domains.

## Methods

### Literature search

Studies were eligible for the present study if EMSs were assessed with a form of the YSQ and a full correlation matrix of all 18 EMSs in the current schema list was reported in the publication. In addition, because correlations between EMSs can be calculated from factor loadings, studies presenting a complete factor or component loading matrix after EFA or principal component analysis (PCA) with orthogonal rotation were included. When oblique rotation was used or a CFA was performed, the factor correlations had to be reported in order to be included in this study. Furthermore, to be eligible for this study publications had to be available from the university's library or digital repositories and be written in English, German, French, Spanish, or a Scandinavian language.

A systematic literature search was conducted on December 10, 2021 (Google Scholar), and on December 11, 2021 (PsycInfo, Medline, EMBASE, and Web of Science). The following key words were used: “Early maladaptive schemas” AND (“factor structure” OR “factor analysis” OR “principal component analysis” OR “intercorrelations”). The search was repeated on May 26, 2022, and more recent results were added. The search results were processed in Zotero, and duplicates were removed before the titles and abstracts of the publications were screened. Finally, full texts were reviewed for tables or figures displaying correlations between YSQ scales or factor loadings. The following information was extracted from the included studies: authors, publication year, publication type (i.e., journal article or thesis), country, sample size, sample type (i.e., clinical, non-clinical, or mixed), mean age and standard deviation of the sample, percentage of female participants, version of the YSQ used, and the data provided (i.e., correlations or factor loadings).

The study was preregistered on https://osf.io/57wyz.

### Analyses

The statistical analyses were conducted in R (v4.2.0) (35). First, correlations between EMSs were calculated for studies that reported factor loadings and factor correlations using the psych package (v2.2.5) (36). Next, all bivariate correlations between EMSs that were computed from factor analyses and reported in the publications were meta-analytically pooled using a univariate random effects approach with the meta package (v5.2-0) (37). Fisher z-transformation of the correlations was applied. To calculate between-study variance, the restricted maximum likelihood estimator was used. Pooled correlations were obtained for the total sample and separately for the clinical and non-clinical subsamples. Deviating from the preregistered protocol, it was not adjusted for score unreliability because reliability is usually reported as Cronbach's alpha, but important assumptions (e.g., tau-equivalence) for using alpha are often not met (38).

The pooled correlation matrices were statistically analyzed using CFA and EFA. The domain models in Table 1 that include all 18 EMSs were tested in a series of CFAs with maximum likelihood estimation in the total sample and in the clinical and non-clinical subsamples using the lavaan package (v0.6-11) (39). Model fit was assessed with the comparative fit index (CFI), the Tucker-Lewis index (TLI), the root mean square error of approximation (RMSEA), and the standardized root mean square residual (SRMR). CFI and TLI values ≥0.95 and RMSEA and SRMR values ≤0.05 are commonly considered to indicate a good model fit, whereas values ≥0.90 (CFI and TLI) and ≤0.08 (RMSEA and SRMR) suggest an acceptable fit (cf) (24).

Further, PCAs with 1–5 factors were conducted to investigate the structure of the pooled correlations between EMSs. The hierarchical structure of the different factor solutions was examined using Goldberg's (40) bass-ackwards method. Oblique rotation (oblimin) was used in all analyses. Parallel analysis, the empirical Kaiser criterium (41), and model fit (SRMR) were used as statistical tools to determine the number of components to be extracted (cf) (42). Further extraction criteria were at least three EMSs with loadings of 0.40 or higher per component and the interpretability of the factors. To investigate the robustness of the chosen component solution, the factor similarities in the clinical and non-clinical samples were calculated. Lorenzo–Seva and Ten Berge (43) suggested that values between 0.85 and 0.94 show that the similarity is fair and that factors with values higher than 0.95 can be considered equal. The psych (v2.2.5) (36) and EFAtools (v0.4.1) (44) packages were used to perform the analyses.

## Results

### Study selection and study characteristics

**Figure 1:**
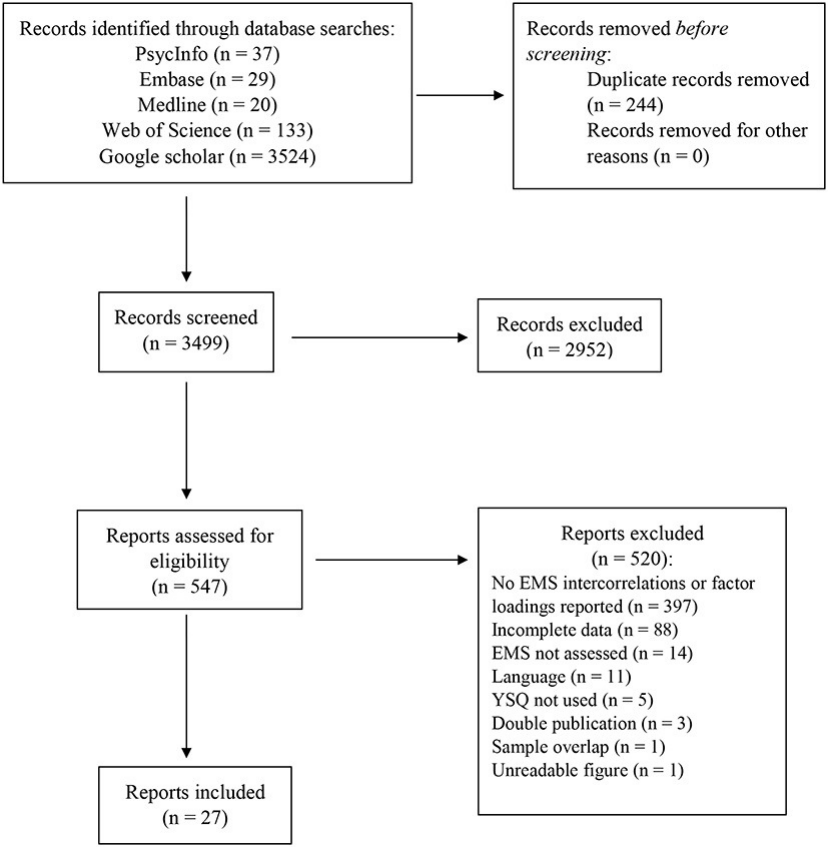
Flowchart of the search and selection procedure.

The identification of studies is shown in Figure 1. A total of 3,499 records were screened, and 547 publications were reviewed in full text. Twenty-seven studies reporting the associations between EMSs in 33 independent samples met the eligibility criteria and were included in this analysis (references can be found in the online Supplementary material). The study characteristics are shown in Table 2. The included studies were published between 2007 (13) and 2022 (21, 50), and 19 publications were journal articles, seven were theses, and one was published as a preprint. Most studies were conducted in Australia (*n* = 4), followed by Italy (*n* = 3), Finland (*n* = 3), Hungary (*n* = 2), and Germany (*n* = 2). The total sample size was 13,958 individuals, including 11,024 non-clinical participants, 1,769 clinical participants, and 1,165 participants from mixed clinical and non-clinical samples. The mean age of the samples ranged from 16.0 to 72.3 years, and on average 64.0% were female. Nineteen studies assessed EMSs with the YSQ-S3, one study used the YSQ-L3, and seven studies administered modified previous versions of the YSQ. Translations of the YSQ were used in 22 studies. Correlations between YSQ scales were provided in 21 publications, while four and two publications reported the results from EFAs and CFAs, respectively.

**Table 2:** Characteristics of the samples included in the meta-analysis.

**References**	**Publication type**	**Country**	***N***	**Sample**	**Mean age in years (SD)**	**% female**	**Instrument**	**Data**
Alfasfos (45)	Thesis	Palestine	200	Non-clinical	22.7 (2.8)	48.5	YSQ-S3	Correlations
Alipan (46)	Thesis	Australia	57	Non-clinical			YSQ-S3	Correlations
	Thesis	Australia	194	Non-clinical			YSQ-S3	Correlations
	Thesis	Australia	67	Non-clinical			YSQ-S3	Correlations
Aloi et al. (25)	Journal article	Italy	1372	Non-clinical	19.5 (2.7)	61.7	YSQ-S3	Correlations
Anttila (47)	Thesis	Finland	306	Mixed	39.0 (14.9)	60.1	extended YSQ-SF	CFA
Askari (48)	Journal article	International	86	Non-clinical	32.2 (9.4)	62.8	YSQ-S3	Correlations
Bach et al. (49)	Journal article	Denmark	567	Mixed	29.4	78.3	YSQ-S3	Factor correlations
Baník et al. (50)	Preprint	Slovakia	270	Non-clinical	40.8 (12.9)	47.4	YSQ-S3	Correlations
Calvete et al. (18)	Journal article	Spain	952	Non-clinical	20.6 (2.8)	54.7	YSQ-S3	Correlations
Csukly et al. (16)	Journal article	Hungary	107	Clinical	41.1 (11.3)	84.1	extended YSQ-L2	EFA
Grutschpalk, (51)	Thesis	Germany	130	Clinical	38.8 (10.8)	0.0	modified YSQ-L2	Correlations
	Thesis	Germany	191	Clinical	36.9 (10.8)	100	modified YSQ-L2	Correlations
Hawke and Provencher (27)	Journal article	Canada	96	Clinical	36.3 (12.7)	66.7	YSQ-S3	Correlations
	Journal article	Canada	973	Non-clinical	26.8 (9.0)	74.6	YSQ-S3	Correlations
Heineck de Souza et al., (52)	Journal article	Brazil	1,050	Non-clinical	30.7 (11.3)	80.7	YSQ-S3	Factor correlations
Jain and Singh (53)	Journal article	India	702	Non-clinical	20.0 (3.9)	51.3	YSQ-S3	Correlations
Kirsner (54)	Thesis	Australia	100	Non-clinical		74.0	YSQ-S3	Correlations
Macik and Macik (21)	Journal article	Polen	2,348	Non-clinical	33.9 (13.0)	54.8	YSQ-S3	Correlations
Munuera et al. (55)	Journal article	France	100	Clinical	40.4 (11.3)	61.0	YSQ-S3	Correlations
Nicol et al. (56)	Journal article	Australia	403	Non-clinical	18.6 (1.7)	66.0	YSQ-S3	Correlations
Panic et al. (57)	Journal article	Serbia	102	Non-clinical	27.5 (9.2)	62.7	YSQ-S3	Correlations
Phillips et al. (58)	Journal article	Australia	94	Non-clinical	72.3 (5.9)	50.0	YSQ-S3	Correlations
Quiñones et al. (59)	Journal article	Chile	292	Mixed	26.3 (11.5)	77.4	YSQ-S3	Correlations
Saariaho et al. (17)	Journal article	Finland	268	Clinical	47.1 (9.3)	53.3	extended YSQ-SF	EFA
	Journal article	Finland	324	Non-clinical	47.3 (9.5)	85.5	extended YSQ-SF	EFA
Saggino et al. (29)	Journal article	Italy	148	Clinical	37.9 (10.4)	35.1	YSQ-L3	CFA
	Journal article	Italy	918	Non-clinical	29.9 (12.6)	56.9	YSQ-L3	CFA
Saritas and Gencöz (15)	Journal article	Turkey	356	Non-clinical	16.0 (0.53)	55.6	YSQ-S3	EFA
Sarparanta (60)	Thesis	Finland	43	Clinical	37.9 (15.5)	67.4	YSQ-L2 extended	Correlations
Trincas et al. (61)	Journal article	Italy	456	Non-clinical	39.9 (5.3)	71.9	YSQ-S3	Correlations
Unoka et al. (13)	Journal article	Hungary	114	Clinical	24.4 (6.2)	100	extended YSQ-L2	EFA
Wichmann (62)	Thesis	Germany	572	Clinical	39.4 (14.2)	76.4	German version of YSQ	Correlations

### Pooled correlations between EMSs

The meta-analytically pooled correlations between EMSs are shown in Table 3. The intercorrelations between EMSs ranged from 0.21 (failure and entitlement) to 0.71 (negativity/pessimism and vulnerability to harm and illness).

**Table 3:** Meta-analytically pooled correlation matrix.

	**ED**	**AB**	**MA**	**SI**	**DS**	**FA**	**DI**	**VH**	**EM**	**SB**	**SS**	**EI**	**US**	**ET**	**IS**	**AS**	**NP**	**PU**
ED	–																	
AB	0.48																	
MA	0.52	0.59																
SI	0.57	0.53	0.60															
DS	0.60	0.58	0.57	0.67														
FA	0.44	0.49	0.46	0.52	0.63													
DI	0.42	0.52	0.47	0.54	0.62	0.66												
VH	0.41	0.55	0.55	0.50	0.53	0.52	0.58											
EM	0.33	0.46	0.42	0.41	0.46	0.46	0.51	0.50										
SB	0.49	0.56	0.52	0.57	0.61	0.60	0.62	0.56	0.57									
SS	0.25	0.36	0.36	0.25	0.25	0.25	0.23	0.32	0.32	0.42								
EI	0.46	0.42	0.49	0.56	0.54	0.45	0.44	0.43	0.37	0.51	0.25							
US	0.25	0.36	0.40	0.35	0.32	0.26	0.26	0.36	0.30	0.36	0.38	0.39						
ET	0.28	0.34	0.42	0.36	0.28	0.21	0.28	0.33	0.29	0.29	0.24	0.31	0.41					
IS	0.38	0.46	0.44	0.47	0.48	0.53	0.54	0.45	0.41	0.52	0.23	0.41	0.27	0.47				
AS	0.29	0.49	0.41	0.35	0.38	0.38	0.42	0.46	0.39	0.48	0.30	0.31	0.41	0.44	0.49			
NP	0.44	0.59	0.61	0.54	0.57	0.53	0.56	0.71	0.47	0.60	0.35	0.51	0.45	0.38	0.52	0.50		
PU	0.36	0.44	0.48	0.46	0.49	0.45	0.44	0.47	0.45	0.49	0.35	0.47	0.52	0.35	0.41	0.39	0.59	–

N = 13,958. AB, Abandonment/instability; AS, Approval-seeking/recognition-seeking; DI, Dependence/incompetence; DS, Defectiveness/shame; ED, Emotional deprivation; EI, Emotional inhibition; EM, Enmeshment/undeveloped self; ET, Entitlement/grandiosity; FA, Failure; IS, Insufficient self-control/self-discipline; MA, Mistrust/abuse; NP, Negativity/pessimism; PU, Punitiveness; SB, Subjugation/invalidation; SI, Social isolation/ alienation; SS, Self-sacrifice; US, Unrelenting standards/hypercriticalness; VH, Vulnerability to harm and illness.

### Confirmatory factor analysis

Global model fit of the different models of EMS organization is displayed in Table 4 for the total sample and the clinical and non-clinical samples. Model tests of the Soygüt et al. (14) and Csukly et al. (16) models showed a non-positive definitive covariance matrix of latent variables due to multicollinearity. The tests of the Macik and Macik (21) model produced a warning that convergence had not been achieved. These models were therefore excluded from Table 4. None of the remaining tested models showed a good fit across all fit indices. As shown in Table 4, models with four factors performed overall slightly better than models with three factors. The best model fit was seen for the (13, 20) models in the clinical sample for which all fit indices indicated an acceptable or good fit.

**Table 4:** Results of CFAs of different proposed models.

**Model**	**Sample**	***χ^2^***	**df**	**CFI**	**TLI**	**RMSEA**	**SRMR**
Bach et al. (19)	Clinical	1443.902	122	0.920	0.899	0.078	0.042
	Non-clinical	10505.644	122	0.903	0.878	0.088	0.049
	Total	11489.948	122	0.917	0.896	0.082	0.042
Calvete et al. (18)	Clinical	1790.766	132	0.899	0.883	0.084	0.049
	Non-clinical	13643.594	132	0.874	0.854	0.096	0.055
	Total	14749.576	132	0.894	0.877	0.089	0.049
Saariaho et al. (17) (2 domains)	Clinical	1976.102	134	0.888	0.872	0.088	0.051
	Non-clinical	16056.351	134	0.851	0.830	0.104	0.058
	Total	17245.822	134	0.876	0.858	0.096	0.051
Saariaho et al. (17) (3 domains)	Clinical	1852.926	132	0.895	0.879	0.086	0.049
	Non-clinical	14107.195	132	0.869	0.849	0.098	0.055
	Total	15590.859	132	0.888	0.870	0.092	0.049
Saritas and Gencöz (15)	Clinical	1719.803	125	0.903	0.881	0.085	0.049
	Non-clinical	11622.713	125	0.893	0.868	0.091	0.053
	Total	13200.542	125	0.905	0.884	0.087	0.047
Unoka et al. (13)	Clinical	1268.837	120	0.930	0.911	0.074	0.042
	Non-clinical	10285.632	120	0.905	0.879	0.088	0.050
	Total	10947.497	120	0.921	0.900	0.080	0.043
Yalcin et al. (20)	Clinical	1475.322	126	0.918	0.900	0.078	0.044
	Non-clinical	12194.446	126	0.887	0.863	0.093	0.055
	Total	13063.370	126	0.906	0.886	0.086	0.047

### Principal component analysis

**Figure 2:**
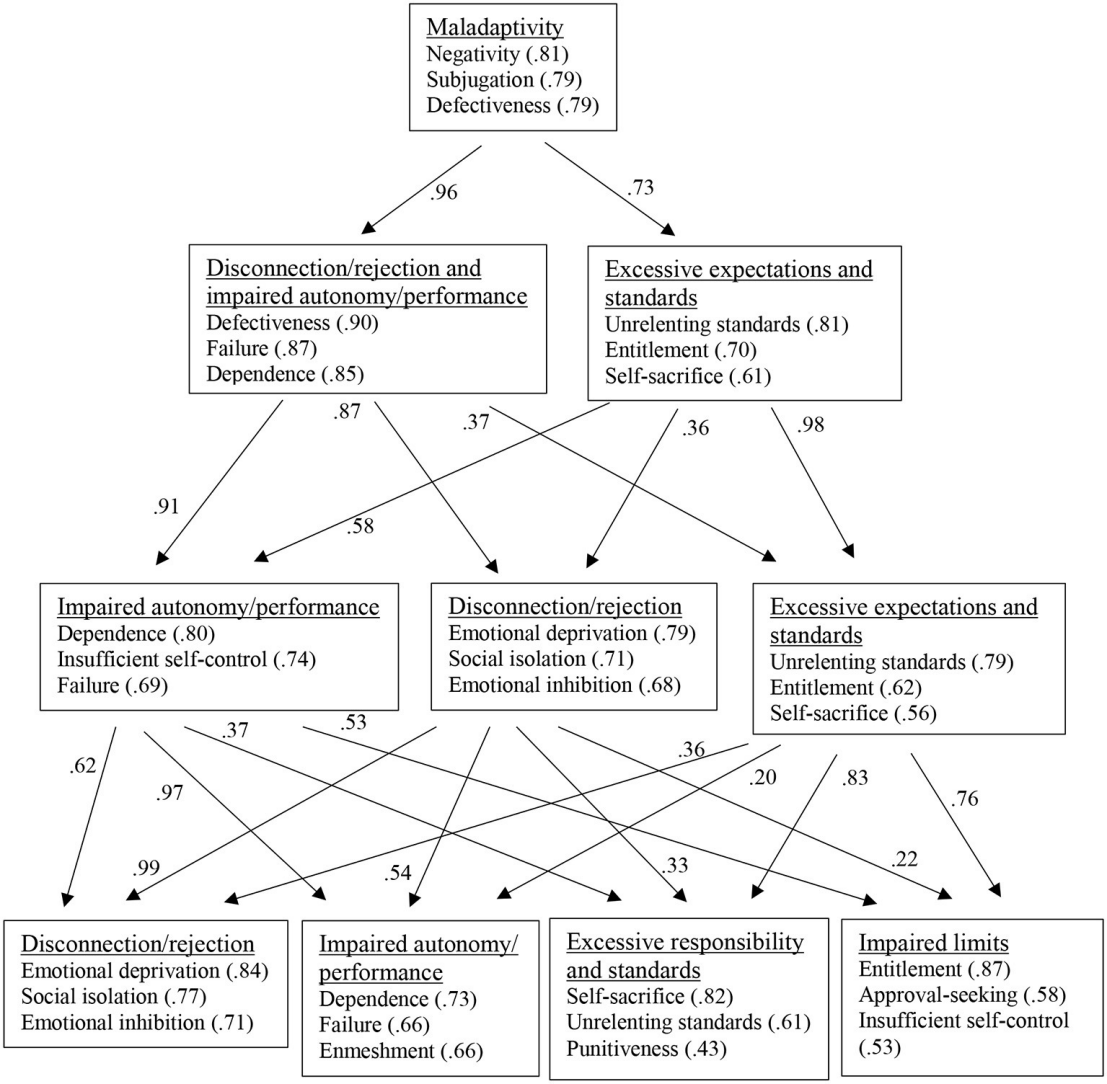
Results of the bass-ackward analysis.

For the total sample, Bartlett's test of sphericity was significant at *p* < 0.05 [$\chi^{2}{}_{\left(153\right)}$ = 137693, *p* < 0.001]. The Kaiser-Meyer-Olkin (KMO) criterion also suggested that the data were suitable for PCA (KMO = 0.95). Figure 2 shows the results from the bass-ackward analysis and provides an overview of the defining EMSs of the components on the different levels of the hierarchy. In the one-component solution, self-sacrifice had the lowest loading (0.47). The component was primarily defined by the negativity/pessimism, the subjugation, and the defectiveness/shame EMSs and was interpreted as a general maladaptivity component. When two components were extracted, the general factor split into a component marked by EMSs from Young's (12) disconnection/rejection and impaired autonomy/performance domains (i.e., defectiveness/shame, failure, dependence/incompetence) and a component that was defined by the unrelenting standards, entitlement, and self-sacrifice EMSs and labeled excessive expectations and standards. In the three-component solution, separate disconnection/rejection (emotional deprivation, social isolation, emotional inhibition) and impaired autonomy/performance components (dependence/incompetence, insufficient self-control, failure) emerged, while the excessive responsibility and standards component largely retained its structure. At the next level, a fourth component emerged that was similar to Young's (12) impaired limits domain and defined by the entitlement, approval-seeking, and insufficient self-control EMSs. In the five-component solution (not shown in Figure 2), the only component loading above 0.40 in the fifth component was self-sacrifice.

Parallel analysis and the empirical Kaiser criterion suggested extracting two components. Solutions with two, three, and four components had at least three EMSs with component loadings larger than 0.40 and showed a slightly better model fit (SRMR = 0.05) than the one-component solution (SRMR = 0.06). Because the four-component solution was more closely aligned to the theoretical model than the solution with three components, it was decided to retain four components. The full pattern matrix is shown in Table 5 (the complete pattern matrices of the one-, two-, three-, and five-component solutions are in Supplementary Table 1 in the online supplementary material). The abandonment/instability and negativity/pessimism EMSs had no component loadings larger than 0.40 but loaded moderately high on impaired autonomy/performance (0.37 and 0.38, respectively). Component correlations ranged from 0.28 (impaired autonomy and performance with excessive responsibility and standards) to 0.54 (impaired autonomy and performance with disconnection/rejection).

**Table 5:** Pattern matrix of the four-factor solution.

	**Disconnection and rejection**	**Impaired autonomy and performance**	**Excessive responsibility and standards**	**Impaired limits**
Emotional deprivation	**0.84**	−0.02	−0.04	−0.04
Abandonment/instability	0.31	0.37	0.19	0.13
Mistrust/abuse	**0.59**	0.06	0.19	0.17
Social isolation/alienation	**0.77**	0.09	−0.03	0.08
Defectiveness/shame	**0.67**	0.32	−0.03	−0.05
Failure	0.29	**0.66**	−0.04	−0.07
Dependence/incompetence	0.20	**0.73**	−0.07	0.04
Vulnerability to harm and illness	0.16	**0.52**	0.21	0.11
Enmeshment/undeveloped self	−0.05	**0.66**	0.28	0.00
Subjugation/invalidation	0.25	**0.57**	0.23	−0.03
Self-sacrifice	−0.04	0.13	**0.82**	−0.11
Emotional inhibition	**0.71**	−0.04	0.12	0.05
Unrelenting standards/ hypercriticalness	0.19	−0.20	**0.61**	0.35
Entitlement/grandiosity	0.11	−0.11	0.02	**0.87**
Insufficient self-control/self-discipline	0.09	**0.50**	−0.20	**0.53**
Approval-seeking/recognition-seeking	−0.20	**0.45**	0.16	**0.58**
Negativity/pessimism	0.28	0.38	0.26	0.19
Punitiveness	0.29	0.14	**0.43**	0.15

Loadings >0.40 in bold.

Two PCAs with oblimin rotation were also performed for the clinical and non-clinical subsamples. Bartlett's test of sphericity was significant at *p* < 0.05 for the clinical [$\chi^{2}{}_{\left(153\right)}$ = 16540.23, *p* < 0.001] and the non-clinical sample [$\chi^{2}{}_{\left(153\right)}$ = 107053.6, *p* < 0.001]. The KMO was 0.95 for both samples, suggesting the suitability of the PCA. Four components were extracted in both samples that were highly similar to the component solution obtained for the total sample (see Supplementary Table 2 in the online supplementary material for the pattern matrices). However, in the clinical sample the defectiveness/shame and insufficient self-control EMSs had their highest loadings on the impaired autonomy and performance component instead of the disconnection and rejection and impaired limits components, respectively. The factor similarities in the two samples were 0.93 (disconnection and rejection), 0.88 (impaired autonomy and performance), 0.95 (excessive responsibility and standards), and 0.96 (impaired limits) suggesting fair to good component congruency.

Taken together, the results from CFAs showed weak support for any of the previously proposed EMS domains. After PCA, four EMS domains were retained that closely resembled the theoretically proposed organization of EMSs.

## Discussion

The purpose of the present study was to use a meta-analytical approach to investigate the higher-order structure of EMSs. Associations between EMSs were obtained from published articles and theses, meta-analytically pooled, and analyzed with CFA and PCA. Results from CFAs of previously suggested and reported EMS organizations showed that no model fit the data well in the total sample. Models with four components were slightly superior to models with two or three components. The Unoka et al. (13) and Yalcin et al. (20) models showed an acceptable to good fit in the clinical subsample. After PCA, four components were retained that closely resembled the EMS organization proposed by Young (12) and Bach et al. (19).

The lack of strong support from CFAs for any of the previously proposed EMS domain models in the present study is consistent with the results of most previous studies that used CFA to test the organization of EMSs [e.g., (21, 24, 30)]. However, these negative findings should not be taken as evidence that EMS domains do not exist. It has been noted that CFA should be used carefully when applied to the examination of the higher-order structure of personality scales because cross-loadings and correlated residuals are common but can result in poor model fit indices if they are not taken into account (63). The models that showed the best fit in the current analyses and had acceptable or near-to acceptable model fits (13, 19, 20) included cross-loadings. However, they still might not have captured the complexity of the EMS interrelations to produce good model fit statistics in the present investigation.

When performing exploratory PCAs on the pooled correlations between EMSs, the statistical criteria (parallel analysis and the empirical Kaiser criterion) suggested extracting two components. The same result has been reported in previous studies (17, 19, 21). However, based on the interpretability of the components, a four-component solution was chosen. This solution was in line with Young's (12) theoretical model and Bach et al.'s (19) classification of the approval-seeking and punitiveness EMSs, which were unclassified in Young's (12) EMS list (see Table 1). However, contrary to Bach et al.'s (19) findings, the previously unclassified negativity/pessimism EMS was more strongly associated with the impaired autonomy domain than the disconnection and rejection domain, suggesting that this EMS is first and foremost related to negative expectations regarding one's ability to influence the outcome of events.

Similar to the studies of Bach et al. (19) and Yalcin et al. (20), some EMSs had high cross-loadings or ambiguous domain affiliations. As in the studies of Bach et al. (19) and Yalcin et al. (20), the insufficient self-control EMS cross-loaded substantially (≥0.40) on the impaired autonomy domain in addition to the primary loading on impaired limits. Likewise, the differences between the primary, secondary, and tertiary loadings of the negativity/pessimism EMS were relatively small. In the bass-ackwards analysis, this EMS defined the general maladaptivity component as in Bach et al. (19), suggesting that the negativity/pessimism EMS captures general distress or demoralization. However, other secondary domain affiliations of the Bach et al. (19) and Yalcin et al. (20) models, e.g., for the punitiveness, vulnerability, enmeshment, and subjugation EMSs, did not emerge in the present analyses. Moreover, the approval-seeking/recognition-seeking EMS had a high cross-loading on impaired autonomy and performance in addition to the primary loading on impaired limits in the present study but not in the two aforementioned studies. The second-highest loading of the abandonment/instability EMS on disconnection and rejection was not much lower than its primary loading on impaired autonomy and performance, which may indicate that this is an interstitial EMS. Notably, the abandonment/instability EMS was initially part of the disconnection and rejection domain (7) but was later moved to the impaired autonomy and performance domain (12).

The four components showed satisfactory congruence in the clinical and non-clinical subsamples. However, small differences in the patterns of component loadings appeared between the two samples. For example, in the clinical sample, defectiveness/shame had a higher loading on the impaired autonomy and performance domain than the disconnection and rejection domain. The insufficient self-control/self-discipline and approval seeking/recognition-seeking EMSs loaded almost equally high on the impaired autonomy and performance and the impaired limits domains. These variations raise the question as to whether some YSQ scales are particularly prone to the effects of mood. This explanation is supported by findings that mood induction affects the reporting of EMSs in the YSQ (64). However, it is also possible that some YSQ scales have different meanings in clinical and non-clinical samples. Rijkeboer and van den Bergh (65) reported factor similarity across a clinical and a non-clinical sample. However, more studies are needed to establish the measurement invariance of the YSQ across clinical and non-clinical populations.

The four-component model was retained because it was most closely aligned to the theoretically proposed groupings of EMSs (12). However, it can be argued that a model with three domains (disconnection and rejection, impaired autonomy and performance, and excessive expectations and standards) represents an equally meaningful organization of EMSs. In the three-component solution, the EMSs of the impaired limits domain loaded on the impaired autonomy and performance domain (approval-seeking/recognition-seeking and insufficient self-control/self-discipline) and a domain that was labeled excessive expectations and standards (entitlement/grandiosity). The approval-seeking/recognition-seeking EMS is defined as an emphasis on approval and recognition from others as the primary source of one's sense of esteem (1). As such, this EMS seems to be conceptually related to the impaired autonomy aspect of the impaired autonomy and performance domain. Similarly, impulsivity and a lack of frustration tolerance as core characteristics of the insufficient self-control/self-discipline EMS will likely be connected with impaired performance. Finally, entitlement/grandiosity refers to the belief that one is superior and entitled to special rights and privileges (1). When three components were extracted, the entitlement/grandiosity EMS clustered together with the self-sacrifice, unrelenting standards/hypercriticalness, and punitiveness EMSs, sharing the theme of unrealistic standards and expectations regarding the behavior of oneself and of others. An advantage of the three-component model is the lack of significant cross-loadings in the present study, i.e., a simple structure. Ultimately, the choice between three or four domains should be guided by theoretical considerations. However, the ambiguous findings in the present as well as in previous studies regarding EMS domains and the high overlap between EMSs (especially negativity/pessimism and vulnerability to harm and illness) also suggest a need to better define the content of EMSs. There is currently work underway to revise the list of EMSs in ST (66), and this will provide the opportunity to improve the definitions and the assessment of EMSs proposed in ST.

A strength of the present investigation is that it draws on a diverse base of samples. On the other hand, the results should be interpreted in the light of some limitations. Given the large number of investigations into EMSs, the number of studies that could be included in the meta-analysis was moderate, mainly because information about the intercorrelations between EMSs was not provided in many publications. Further, to reduce heterogeneity only studies that used a form of the YSQ to assess EMSs were included. However, the equivalence of the different versions of the YSQ is uncertain, especially with regard to the most recently added EMSs. Some researchers have developed and used their own scales for the approval-seeking/recognition-seeking, negativity/pessimism, and punitiveness EMSs [e.g., (17)], but the convergent validity of these scales is unclear. In addition, the YSQ exists in long and short forms, which assess EMSs at different levels of detail. When long forms of the YSQ have been factor-analytically examined, EMS scales sometimes split into two different EMSs, especially the emotional inhibition and punitiveness scales (20, 67). Finally, the coding of studies and the extraction of data was performed by only one researcher, which increases the risk of errors.

In conclusion, the results of the present investigation support the organization of EMSs in the four domains proposed by Young (12) and Bach et al. (19), except for the negativity/pessimism EMS that was affiliated with the impaired autonomy and performance domain rather than the disconnection and rejection domain. However, a three-domain model showed a simpler structure. The results suggest a need for further theoretical and empirical clarification of the higher-order structure of EMS.

## Author contributions

The author confirms being the sole contributor of this work and has approved it for publication.

## Conflict of interest

The author declares that the research was conducted in the absence of any commercial or financial relationships that could be construed as a potential conflict of interest.

## Publisher's note

All claims expressed in this article are solely those of the authors and do not necessarily represent those of their affiliated organizations, or those of the publisher, the editors and the reviewers. Any product that may be evaluated in this article, or claim that may be made by its manufacturer, is not guaranteed or endorsed by the publisher.
