# Supplementary material for: The higher-order structure of early maladaptive schemas: A meta-analytical approach
Source: Front Psychiatry. 2022 Dec 1;13:1053927. doi: 10.3389/fpsyt.2022.1053927 (PMC9753131; doi:10.3389/fpsyt.2022.1053927)
Supplement: Supplementary file 1 [file Data_Sheet_1.docx]

**Supplementary online material**

Studies included in the meta-analysis

Alfasfos, L. (2009). *The early maladaptive schemas and their correlations with the psychiatric symptoms and the personality accentuations for palestinian students* [Doctoral dissertation]. Hamburg University.

Alipan, A. (2020). *A Mixed Methods Investigation of Cyberbullying Behaviours, Early Maladaptive Schemas, Coping Styles, and Psychological Outcomes in Emerging Adults* [Doctoral dissertation]. Hawthorn at Swinburne University of Technology.

Aloi, M., Rania, M., Sacco, R., Basile, B., & Segura-Garcia, C. (2020). The Young Schema Questionnaire Short Form 3 (YSQ-S3): Does the new four-domains model show the best fit? *Anales de Psicología/Annals of Psychology*, *36*(2), 254-261. https://doi.org/10.6018/analesps.36.2.343461

Anttila, N. (2019). *The factor structure of the revised maladaptive schema domains and their role as cognitive vulnerabilities between childhood maltreatment and depressive symptoms* [Master’s thesis]. University of Eastern Finland.

Askari, I. (2019). Early maladaptive schemas and cognitive-behavioral aspect of anger: Schema model perspective. *Journal of Rational-Emotive & Cognitive-Behavior Therapy*, *37*(3), 262-283. https://doi.org/10.1007/s10942-018-0311-9

Bach, B., Simonsen, E., Christoffersen, P., & Kriston, L. (2017). The Young Schema Questionnaire 3 Short Form (YSQ-S3): Psychometric properties and association with personality disorders in a Danish mixed sample. *European Journal of Psychological Assessment*, *33*(2), 134-143. https://doi.org/http://dx.doi.org/10.1027/1015-5759/a000272

Baník, G., Vargová, L., & Zibrínová, Ľ. (2022). Early maladaptive schemas, depression and post-traumatic stress disorder in a trauma-exposed sample: A correlation, regression and network perspective. https://doi.org/10.31234/osf.io/6fhds

Calvete, E., Orue, I., & González-Diez, Z. (2013). An examination of the structure and stability of early maladaptive schemas by means of the Young Schema Questionnaire-3. *European Journal of Psychological Assessment*, *29*(4), 283-290. https://doi.org/10.1027/1015-5759/a000158

Csukly, G., Telek, R., Filipovits, D., Takács, B., Unoka, Z., & Simon, L. (2011). What is the relationship between the recognition of emotions and core beliefs: Associations between the recognition of emotions in facial expressions and the maladaptive schemas in depressed patients. *Journal of Behavior Therapy and Experimental Psychiatry*, *42*(1), 129-137. https://doi.org/10.1016/j.jbtep.2010.08.003

Grutschpalk, J. (2008). *Diagnostik im Rahmen der Schematherapie unter besonderer Berücksichtigung der Persönlichkeitsakzentuierungen* [Doctoral dissertation]. Hamburg University.

Hawke, L. D., & Provencher, M. D. (2012). The Canadian French Young Schema Questionnaire: Confirmatory factor analysis and validation in clinical and nonclinical samples. *Canadian Journal of Behavioural Science/Revue canadienne des sciences du comportement*, *44*(1), 40-49. https://doi.org/10.1037/a0026197

Heineck de Souza, L., Eduarda Anawate Muniz Tavares, M., de Lara Machado, W., & da Silva Oliveira, M. (2022). Evidence of validity for the Brazilian version of the Young Schema Questionnaire—Short Form (YSQ-S3). *Trends in Psychology*, *30*(2), 225-241. https://doi.org/10.1007/s43076-021-00104-z

Jain, A., & Singh, K. (2019). Validation of the Young Schema Questionnaire - Short Form 3 in Indian population. *Psychological Studies, 64*(2), 118-130. https://doi.org/10.1007/s12646-019-00493-3

Kirsner, J. (2018). *Early maladaptive schemas associated with performance anxiety aetiology and phenomenology in classically-trained musicians* [Master’s thesis]. University of Melbourne

Mącik, D., & Mącik, R. (2022). Are four maladaptive schema domains a better option than five? Recommendations based on comparison of the latent structure of schemas on a large group of healthy adults. *Behavioural and Cognitive Psychotherapy*, *50*(3), 334-344. https://doi.org/10.1017/S1352465821000539

Munuera, C., Roux, P., Weil, F., Passerieux, C., & M'Bailara, K. (2020). Determinants of the remission heterogeneity in bipolar disorders: The importance of early maladaptive schemas (EMS). *Journal of Affective Disorders*, *277*, 857-868. https://doi.org/10.1016/j.jad.2020.08.079

Nicol, A., Mak, A. S., Murray, K., & Kavanagh, P. S. (2021). Early maladaptive schemas in young people who self-injure. *Journal of Clinical Psychology*, *77*(7), 1745-1762. https://doi.org/10.1002/jclp.23172

Panic, D., Stanojevic, T. S., & Pesic, M. H. (2016). Early maladaptive schemas among psychosomatic prone individuals. *Ceskoslovenska psychologie*, *60*(1), 1-12.

Phillips, K., Brockman, R., Bailey, P. E., & Kneebone, I. I. (2019). Young Schema Questionnaire–Short Form Version 3 (YSQ-S3): Preliminary validation in older adults. *Aging & Mental Health*, *23*(1), 140-147. https://doi.org/10.1080/13607863.2017.1396579

Quiñones, Á., Ramírez, P., Cid, J., Melipillán, R., Ugarte, C., & Florenzano, R. (2018). Cuestionario de esquemas de Young CEY-S3: Propiedades psicométricas en una muestra chilena mixta. *Terapia psicológica*, *36*(3), 144-155. https://doi.org/10.4067/S0718-48082018000300144

Saggino, A., Balsamo, M., Carlucci, L., Cavalletti, V., Sergi, M. R., da Fermo, G., Dettore, D., Marsigli, N., Petruccelli, I., Pizzo, S., & Tommasi, M. (2018). Psychometric properties of the Italian version of the Young Schema Questionnaire L-3: Preliminary results. *Frontiers in Psychology*, *9*. https://doi.org/10.3389/fpsyg.2018.00312

Saritaş, D., & Gençöz, T. (2011). Psychometric properties of “Young Schema Questionnaire–Third version” in a Turkish adolescent sample. *Journal of Cognitive and Behavioral Psychotherapies*, *11*(1), 83-96.

Sarparanta, S. (2015). *Are early maladaptive schemas associated with borderline personality disorder symptomatology among depressed adult inpatients?* [Master’s Thesis]. University of Helsinki.

Saariaho, T., Saariaho, A., Karila, I., & Joukamaa, M. (2012). Early maladaptive schema factors, chronic pain and depressiveness: A study with 271 chronic pain patients and 331 control participants. *Clinical Psychology & Psychotherapy*, *19*(3), 214-223. https://doi.org/10.1002/cpp.737

Trincas, R., Ottaviani, C., Couyoumdjian, A., Tenore, K., Spitoni, G., & Mancini, F. (2014). Specific dysphoric symptoms are predicted by early maladaptive schemas. *The Scientific World Journal*. https://doi.org/10.1155/2014/231965

Unoka, Z., Tolgyes, T., & Czobor, P. (2007). Early maladaptive schemas and body mass index in subgroups of eating disorders: A differential association. *Comprehensive Psychiatry*, *48*(2), 199-204. https://doi.org/10.1016/j.comppsych.2006.09.002

Wichmann, G. (2012). *Eine Überprüfung der Psychometrischen Qualität desYoung-Schema-Questionnaire (YSQ-S2)* [Master’s Thesis]. Humboldt-University Berlin.

Table S1: Pattern matrices with one, two, three, and five components

|  | One component |  | Two components | |  | Three components | | |  | Five components | | | | |
| --- | --- | --- | --- | --- | --- | --- | --- | --- | --- | --- | --- | --- | --- | --- |
|  | C1 |  | C1 | C2 |  | C1 | C2 | C3 |  | C1 | C2 | C3 | C4 | C5 |
| Emotional deprivation | **.65** |  | **.74** | -.09 |  | -.01 | **.79** | .02 |  | -.04 | **.87** | -.11 | .04 | .12 |
| Abandonment/ instability | **.75** |  | **.61** | .22 |  | **.45** | .29 | .18 |  | .31 | .36 | -.07 | .20 | .31 |
| Mistrust/abuse | **.75** |  | **.57** | .28 |  | .14 | **.55** | .31 |  | .04 | **.56** | .13 | .19 | .20 |
| Social isolation/ alienation | **.75** |  | **.78** | .01 |  | .15 | **.71** | .07 |  | .14 | **.68** | .14 | .08 | -.06 |
| Defectiveness/shame | **.79** |  | **.90** | -.11 |  | .35 | **.63** | -.07 |  | .38 | **.57** | .10 | -.04 | -.04 |
| Failure | **.73** |  | **.87** | -.15 |  | **.69** | .27 | -.20 |  | **.73** | .19 | .06 | -.06 | -.06 |
| Dependence/incompetence | **.75** |  | **.85** | -.10 |  | **.80** | .16 | -.18 |  | **.77** | .12 | .02 | .05 | -.07 |
| Vulnerability to harm and illness | **.75** |  | **.61** | .22 |  | **.60** | .15 | .14 |  | **.54** | .08 | .18 | .10 | .14 |
| Enmeshment/undeveloped self | **.66** |  | **.54** | .19 |  | **.69** | -.02 | .08 |  | **.66** | -.10 | .11 | .01 | .24 |
| Subjugation/invalidation | **.79** |  | **.73** | .12 |  | **.60** | .26 | .06 |  | **.57** | .21 | .06 | .00 | .25 |
| Self-sacrifice | **.47** |  | .03 | **.61** |  | .07 | .08 | **.56** |  | .00 | .06 | .06 | -.04 | **.89** |
| Emotional inhibition | **.67** |  | **.60** | .12 |  | .00 | **.68** | .20 |  | .08 | **.49** | **.48** | -.06 | -.14 |
| Unrelenting standards/ hypercriticalness | **.55** |  | -.04 | **.81** |  | -.10 | .20 | **.79** |  | -.13 | -.04 | **.79** | .16 | .16 |
| Entitlement/grandiosity | **.52** |  | .01 | **.70** |  | .21 | -.05 | **.62** |  | -.18 | .13 | .14 | **.84** | -.02 |
| Insufficient self-control/self-discipline | **.68** |  | **.56** | .20 |  | **.74** | -.04 | .08 |  | **.48** | .09 | -.07 | **.55** | -.13 |
| Approval-seeking/ recognition-seeking | **.63** |  | .23 | **.56** |  | **.68** | -.28 | .39 |  | .37 | -.16 | .03 | **.60** | .18 |
| Negativity/pessimism | **.81** |  | **.59** | .33 |  | **.48** | .25 | .27 |  | **.43** | .14 | .35 | .13 | .08 |
| Punitiveness | **.69** |  | .36 | **.47** |  | .20 | .30 | **.44** |  | .26 | .01 | **.73** | -.02 | .00 |

Loadings > .40 in bold.

Table S2: Pattern matrices of the four-factor solution in the clinical and nonclinical subsamples

|  | Clinical | | | |  | Nonclinical | | | |
| --- | --- | --- | --- | --- | --- | --- | --- | --- | --- |
|  | Disconnection and rejection | Impaired autonomy and performance | Excessive responsibility and standards | Impaired limits |  | Disconnection and rejection | Impaired autonomy and performance | Excessive responsibility and standards | Impaired limits |
| Emotional deprivation | **.87** | -.07 | .02 | -.01 |  | **.81** | .03 | -.03 | -.08 |
| Abandonment/ instability | .31 | .31 | .15 | .25 |  | .32 | .29 | .27 | .18 |
| Mistrust/abuse | **.61** | .09 | .19 | .18 |  | **.57** | -.01 | .21 | .19 |
| Social isolation/ alienation | **.55** | .37 | -.06 | .13 |  | **.86** | -.01 | -.06 | .04 |
| Defectiveness/shame | **.44** | **.54** | .02 | .01 |  | **.76** | .26 | -.05 | -.04 |
| Failure | .16 | **.77** | -.06 | -.07 |  | .37 | **.57** | .02 | .04 |
| Dependence/ incompetence | .10 | **.81** | -.09 | .02 |  | .26 | **.63** | .02 | .16 |
| Vulnerability to harm and illness | .15 | **.41** | .25 | .15 |  | .23 | **.41** | .26 | .22 |
| Enmeshment/undeveloped self | -.21 | **.79** | .16 | -.03 |  | .01 | **.53** | .36 | .11 |
| Subjugation/invalidation | .13 | **.68** | .23 | -.06 |  | .33 | **.46** | .29 | .06 |
| Self-sacrifice | .07 | -.02 | **.84** | -.13 |  | -.08 | .08 | **.88** | -.08 |
| Emotional inhibition | **.47** | .28 | .14 | .04 |  | **.78** | -.12 | .06 | .02 |
| Unrelenting standards/ hypercriticalness | -.03 | .05 | **.64** | .32 |  | .23 | **-.41** | **.52** | .34 |
| Entitlement/grandiosity | .08 | -.13 | .02 | **.91** |  | .06 | -.21 | -.02 | **.86** |
| Insufficient self-control/self-discipline | .08 | **.50** | -.26 | **.48** |  | .09 | .37 | -.16 | **.65** |
| Approval-seeking/ recognition-seeking | -.23 | **.45** | .22 | **.46** |  | -.14 | .21 | .11 | **.76** |
| Negativity/pessimism | .19 | **.43** | .28 | .20 |  | .34 | .25 | .30 | .24 |
| Punitiveness | .20 | .22 | **.44** | .23 |  | .33 | -.03 | **.42** | .16 |

Loadings > .40 in bold.
